# Infrastructure expansion challenges sustainable development in Papua New Guinea

**DOI:** 10.1371/journal.pone.0219408

**Published:** 2019-07-24

**Authors:** Mohammed Alamgir, Sean Sloan, Mason J. Campbell, Jayden Engert, Regina Kiele, Gabriel Porolak, Thomas Mutton, Ambroise Brenier, Pierre L. Ibisch, William F. Laurance

**Affiliations:** 1 Centre for Tropical Environmental and Sustainability Science, and College of Science and Engineering, James Cook University, Cairns, Queensland, Australia; 2 Remote Sensing Centre, School of Natural and Physical Sciences, The University of Papua New Guinea, Port Moresby, Papua New Guinea; 3 Wildlife Conservation Society, Papua New Guinea Program, Goroka, Eastern Highlands Province, Papua New Guinea; 4 Centre for Econics and Ecosystem Management, Eberswalde University for Sustainable Development, Eberswalde, Germany; Centre for Cellular and Molecular Biology, INDIA

## Abstract

The island of New Guinea hosts the third largest expanse of tropical rainforest on the planet. Papua New Guinea—comprising the eastern half of the island—plans to nearly double its national road network (from 8,700 to 15,000 km) over the next three years, to spur economic growth. We assessed these plans using fine-scale biophysical and environmental data. We identified numerous environmental and socioeconomic risks associated with these projects, including the dissection of 54 critical biodiversity habitats and diminished forest connectivity across large expanses of the island. Key habitats of globally endangered species including Goodfellow’s tree-kangaroo (*Dendrolagus goodfellowi*), Matchie’s tree kangaroo (*D*. *matschiei*), and several birds of paradise would also be bisected by roads and opened up to logging, hunting, and habitat conversion. Many planned roads would traverse rainforests and carbon-rich peatlands, contradicting Papua New Guinea’s international commitments to promote low-carbon development and forest conservation for climate-change mitigation. Planned roads would also create new deforestation hotspots via rapid expansion of logging, mining, and oil-palm plantations. Our study suggests that several planned road segments in steep and high-rainfall terrain would be extremely expensive in terms of construction and maintenance costs. This would create unanticipated economic challenges and public debt. The net environmental, social, and economic risks of several planned projects—such as the Epo-Kikori link, Madang-Baiyer link, Wau-Malalaua link, and some other planned projects in the Western and East Sepik Provinces—could easily outstrip their overall benefits. Such projects should be reconsidered under broader environmental, economic, and social grounds, rather than short-term economic considerations.

## Introduction

Papua New Guinea (PNG) sustains one of the few remaining strongholds for tropical forest on Earth. It contains about 328,000 km^2^ tropical forests [1]—among the largest tropical forest estates of any nation. PNG forests have exceptionally high species endemism, with many species still not scientifically described [1], and are some of the most carbon-rich forests on the planet. Many of the 8 million inhabitants of PNG rely on these forests either directly or indirectly for their livelihood or subsistence incomes [1, 2].

The historical trends of forest management in PNG are attributed to forest exploitation in several forms—commercial logging, large forest concessions for agricultural development, large-scale mining, expanding oil palm plantations, and a long history of swidden farming by its indigenous peoples [1, 3, 4]. Via these trends, PNG has lost about 3,750 km^2^ of rainforest since 2002, and a further 7,700 km^2^ is experiencing various forms of degradation [1].

Road development in forest frontiers leads to several primary and secondary impacts. The primary impacts include local deforestation for road construction, some greenhouse gas emissions, local changes in abiotic and biotic conditions caused by edge effects, increased erosion and stream sedimentation, and wildlife roadkill [5–8]. The secondary impacts include incursions of hunters and poachers; legal and illegal logging, mining, forest fires, and land encroachment; landslides and impeded water flows; invasions of disturbance-favoring species; and land speculation; among other impacts [5, 8, 9].

**Fig 1:**
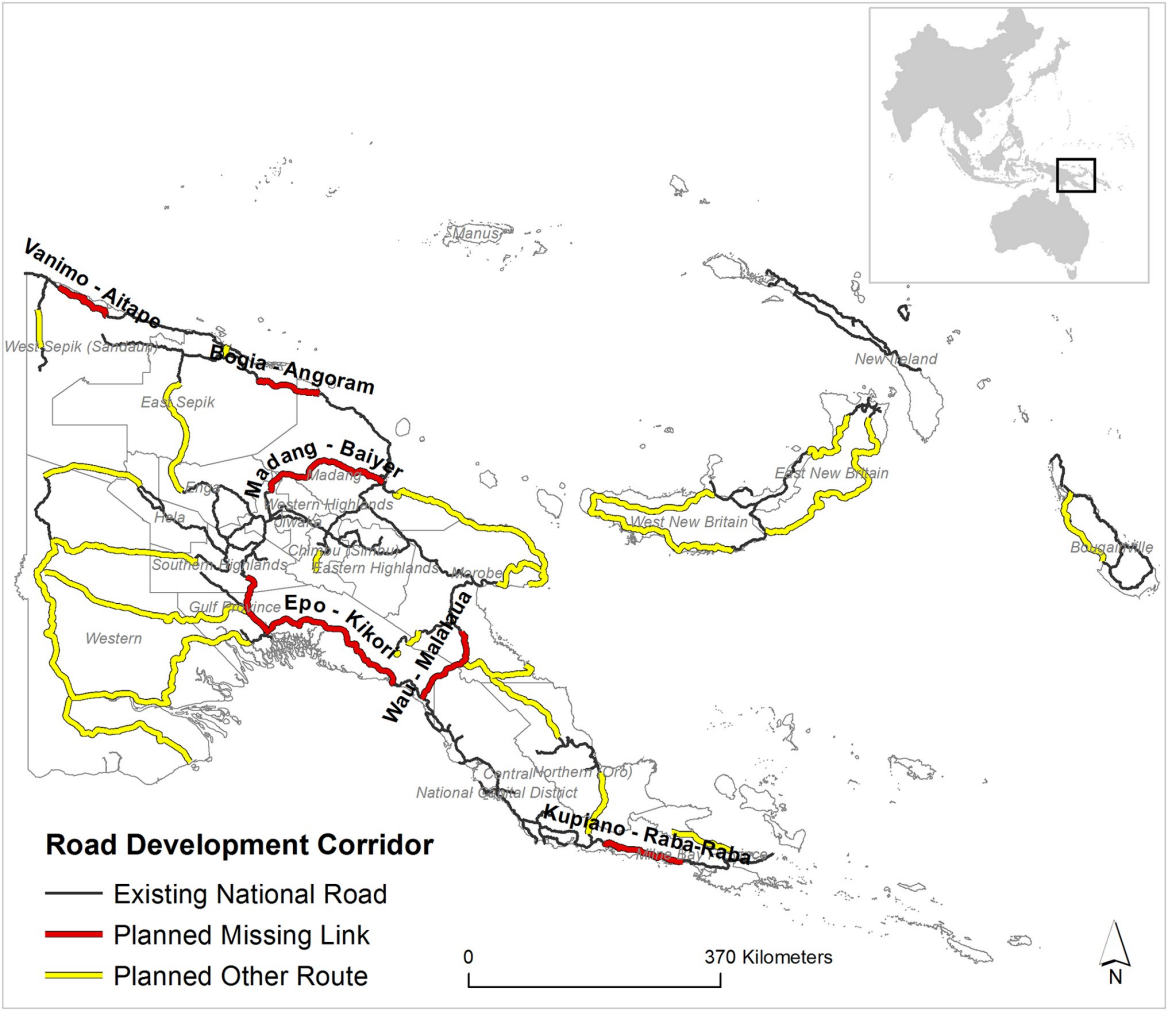
Road development corridor in Papua New Guinea. Existing national roads, planned missing links, and other planned road-route data (from [2]).

PNG plans to dramatically increase its current road extent in the coming years to promote economic development (Fig 1). Overall, the current ~8,700 km of national road length will be increased to 15,000 km by the end of 2022. This development is planned under two different umbrella projects—“Missing Links” and “Other Planned Roads” [2, 10]. The missing link projects are articulated as national priorities and are planned to connect the current disjoined national roads to build economic-development corridors.

The missing links projects are still evolving. However, six priority missing-link projects are listed in the Medium Term Development Plan for PNG [2]: Vanimo—Aitape, 105 km; Bogia—Angoram, 101 km; Wau—Malalaua, 132 km; Kupiano—Raba-Raba, 142 km; Epo—Kikori 105 km (partial); and Madang-Baiyer, 263 km. The other planned projects particularly focus on construction of national roads in areas where there are few roads, such as western PNG, New Britain Province, and the Autonomous Region of Bouganville [2] (Fig 1).

The road expansion agenda is intended to enhance near-term economic growth and regional connectivity in PNG. However, the environmental and broader societal impacts of this massive infrastructure expansion are still unknown. Here we investigate the potential impacts of planned road expansion on overall environmental, economic, and social integrity in the region, specifically on forest connectivity, biodiversity, carbon storage, deforestation and industrial development. We conclude by discussing different options that could improve the road expansion process in PNG, facilitating economic, environmental, and social benefits while reducing environmental degradation.

## Methods

### Planned road expansion

We obtained the latest map of planned road network development from the Medium Term Development Plan III 2018–2022, of the PNG government [2]. The map contained the planned roads—‘missing links’ and ‘other routes’—along with existing national roads across PNG. We digitized all these roads in the GIS interface following Alamgir et al. [11] and Sloan et al. [12]. The digitization procedure entailed geo-referencing the map and then tracing the road layers in a GIS. Subsequently, we retraced the digitized GIS layer in Google Earth aiming to achieve maximum accuracy with reference to alignment of existing roads. The estimated locational error of the final digitized features was <200 m relative to Google Earth imagery.

### Landscape connectivity and deforestation

Landscape connectivity assesses the linkages between different forest patches in a landscape. To evaluate the potential impacts of planned roads on current landscape connectivity of forests across PNG, we conducted a morphological spatial pattern analysis (MSPA). The MSPA analysis was performed using Graphical User Interface for the Description of Image Objects and their Shapes (Guidos Toolbox 2.6 version 4) [13, 14]. The MSPA analysis segmented the forests of PNG into distinct forest-landscape elements, namely core forest patches (forest ≥ 600 m from the nearest forest edge), edge forest (forest < 600 m from an edge), and connectivity forest (forest corridors connecting different core-forest patches or different sections of a core-forest patch). Subsequently, we identified areas where planned roads will intersect distinct forest-landscape elements, particularly core forests and connectivity forests. In MSPA, we used forest data from the updated (v.1.5.) annual 30-m Landsat classifications of Hansen et al. [15]. Areas with <50 percent tree cover were excluded from the analysis, considering the tropical forest attributes in the region. Data were resampled at a 200-m resolution for the purpose of processing. MSPA capture changes to both fragmentation and connectivity across a forest landscape while retaining a spatially explicit focus on critical forest patches [11, 14], unlike other approaches in fragmentation analysis. Our 600-m threshold defining edge effects is taken as a conservative indicator of potential edge effects on forest patches; a 1000-m threshold has been widely used in other road-impact research [16, 17]. In fact, edge effects can extend from a few meters to several kilometers from the forest periphery to its interior [18] and include both abiotic and biotic components of the forested ecosystems. Some biotic effects include unwanted changes of plant and animal communities such as increased abundance of lianas and rattans, and decreased abundance of mammals and birds [7, 18–21]. Some abiotic effects include increased wind disturbances, reduced soil moisture, and increased air temperatures [18]. Edge effects may become weaker with time if the forest is subsequently undisturbed, but in the case of road building, edge effects are likely to become worse with time as road development facilitates further disturbances, such as the expansion of agriculture, settlements, mining, logging, and the construction of secondary and tertiary roads.

We calculated average deforestation in PNG since 2000 within a 3-km radius of a pixel following Sloan et al. [22]. For this process, we used deforestation data between 2000 and 2017 from the updated (v.1.5.) annual 30-m Landsat classifications of Hansen et al. [15]. Data were resampled to 200-m resolution for consistency with our other analyses.

### Protected areas, peatlands, slope, and mining concessions

To spatially evaluate the planned roads with respect to conservation areas, peatlands, and industrial development, we overlaid the planned routes over protected areas, biodiversity-conservation priority areas, peatlands, intact forest, slope data, and mining-concession areas across PNG. Protected area-data were obtained from the World Database on Protected Areas [23] and complemented by the protected areas map of PNG [24] and the manual addition of a recently declared protected area [25]. The protected areas are listed under various forest designations, including national parks and wildlife management areas (WMAs). In WMAs, local communities are allowed regulated hunting and other forms of land uses because land is customarily owned. Biodiversity-conservation priority areas designate an area (forest/wetland) with significant biodiversity values that require immediate conservation attention, presumably candidates for future protected areas (if not currently protected). The areas are classified into ‘very high priority’ and ‘high priority’ considering overall biodiversity values and conservation significance, integrating biophysical data (such as climate and vegetation) and expert opinions [26]. These biodiversity-conservation priority areas [26] data were obtained from the Remote Sensing Centre, University of Papua New Guinea. The peatlands data for PNG were extracted from the latest tropical wetlands and peatlands data [27, 28], which is considered more robust than other available peatland data (e.g. Page et al. [29]). We calculated slope across PNG from widely used Global Multi-resolution Terrain Elevation Data 2010 (GMTED2010) at 250-m spatial resolution [30]. We obtained the rainfall data for the wettest quarter for PNG at 1-km^2^ resolution [31]. We evaluated the planned-roads profile with respect to slope and rainfall in the wettest quarter of the year, which is when the likelihood of landslides is highest. Mining-concession data for PNG were digitized in GIS from a map from the PNG mining cadastre portal [32]. The map contained the updated mining-concession polygons with the current status of each site, such as active mining or application for new mining.

## Results

### Planned roads will penetrate biodiversity-conservation areas

**Fig 2:**
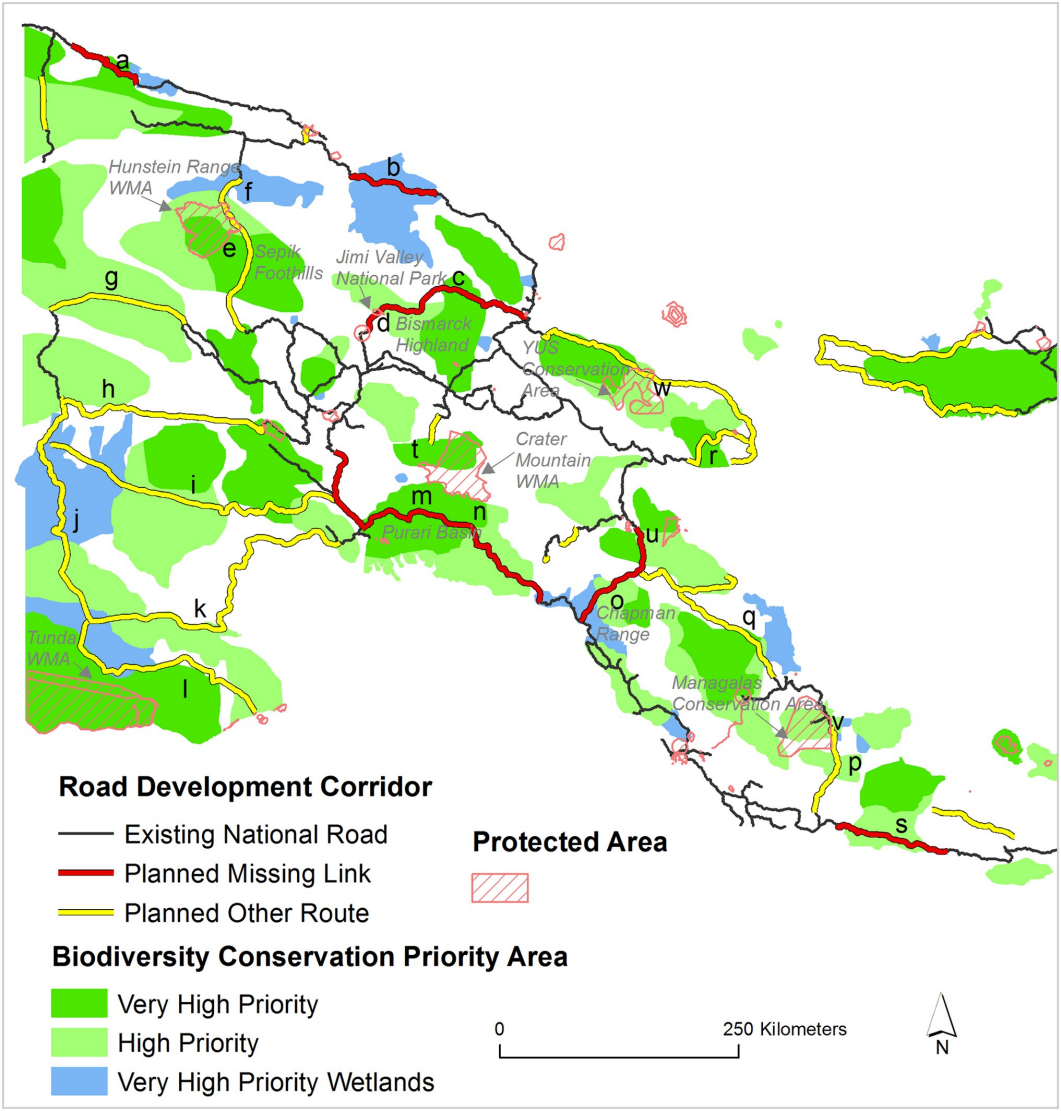
Planned roads penetrating into significant biodiversity-conservation areas. Different letters indicate areas where roads, if completed as planned, would dissect protected areas and biodiversity conservation-priority areas.

Planned roads development are set to dissect 45 biodiversity-conservation priority areas and 9 protected areas in PNG (Fig 2). Additionally, 8 more protected areas are within <25 km of the planned road development. These biologically important areas are currently free from major road incursion and will be directly and indirectly impacted by planned road development.

Notably, 111 km of the Madang-Baiyer missing link will traverse the core of the Jimi Valley National Park and biodiversity conservation priority areas in the Bismarck Highlands (Fig 2, marked ‘c’ and ‘d’)—a home of many species of birds of paradise and the endangered Goodfellow’s tree kangaroo (*Dendrolagus goodfellowi)* [33]. Nearly 150 km of a planned road project in East Sepik foothills (Fig 2, ‘marked e’) will dissect two priority biodiversity-conservation areas and the Hunstein Range Wildlife Management Area, one of the largest lowland rainforest protected areas in PNG with both exceptional biodiversity and indigenous-cultural values. Nearly 240 km of the Epo-Kikori missing link will divide two priority biodiversity-conservation areas in Purari Basin (Fig 2, marked ‘m’ and ‘n’) and will be within <25 km of Crater Mountain and Neiru Wildlife Wanagement Areas. Both of these wildlife management areas are managed by local indigenous peoples. In fact, the Crater Mountain Wildlife Management Area is the second-largest terrestrial protected area in PNG. Almost 95 km of the Wau-Malalaua missing link is set to divide four biodiversity-conservation areas in Chapman Range and East Gulf, and three protected areas—Mt. Kinadi, Mc Adams, and Kamiali Wildlife Management Areas—are <25 km from the planned link. The forest in this region is relatively undisturbed and forms a very large block of rainforest in southeastern PNG.

The planned road project in Morobe province (Fig 2, marked ‘w’) will dissect the YUS conservation area—an intact rainforest landscape with high diversity of flora and fauna and home to the endangered Matchie’s tree kangaroo (*Dendrolagus matschiei*) [34], the range-restricted emperor bird-of-paradise (*Paradisaea guilielmi*) and the Huon astrapia (*Astrapia rothschildi*). The planned road in southeastern PNG is set to dissect the newest protected area in PNG, the Managalas Conservation Area (Fig 2, marked ‘v’). This conservation area is the largest protected area in PNG, consisting of 3,600 km^2^ of largely intact rainforest [25].

### Losses of forest connectivity

**Fig 3:**
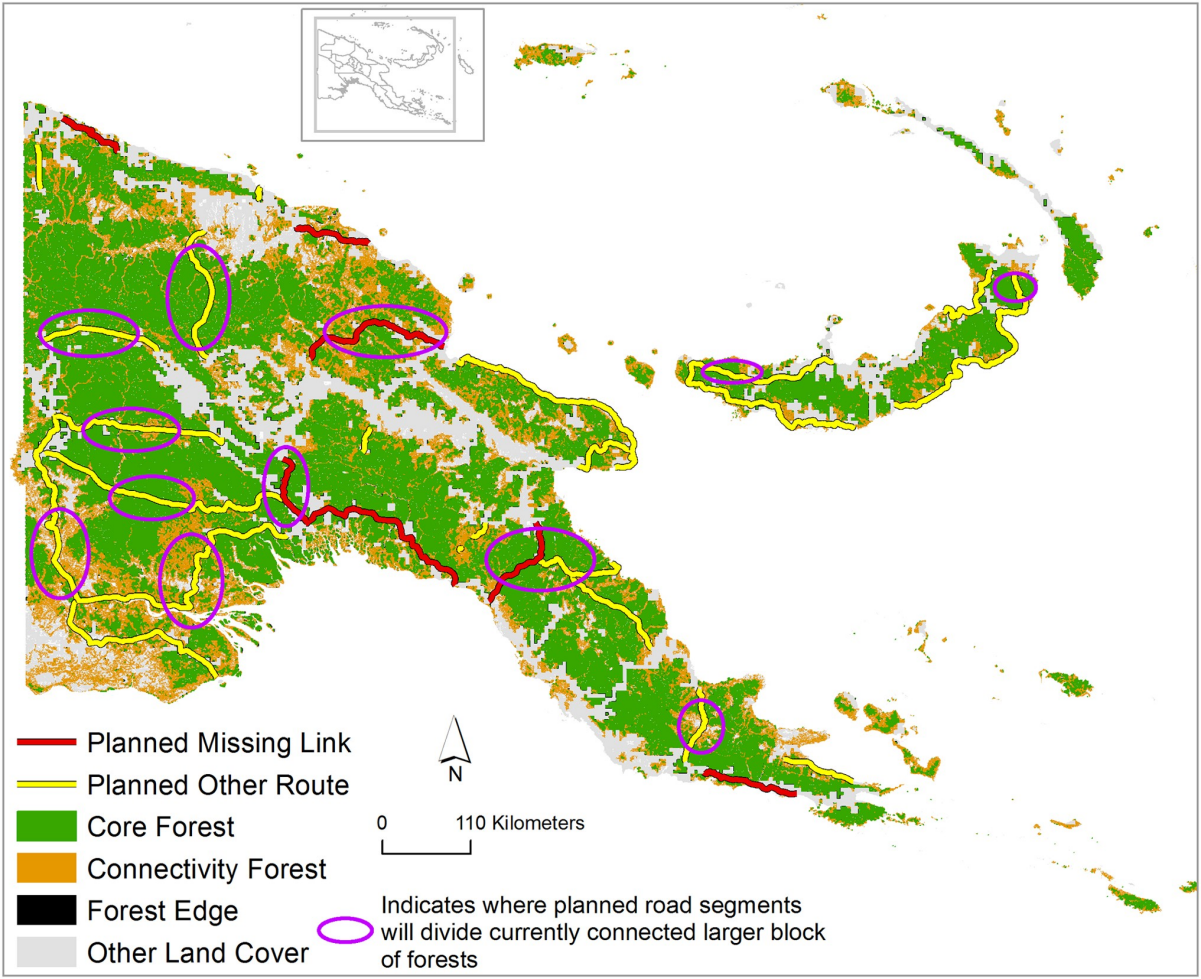
Planned infrastructure expansion and forest spatial pattern in Papua New Guinea. “Core forest” indicates relatively intact forest patches ≥ 600 m from the nearest forest edge; a “connectivity forest” is a connecting pathway between two sections of a core forest or different core forest; “edge forest” is <600 m from the nearest forest edge.

Planned road development in PNG will substantially reduce current forest connectivity by decreasing both connectivity forest and core forest (Fig 3). Connectivity forests are crucial to maintain connectivity between different forest patches in a landscape whereas core forests maintain unique forested habitat for biodiversity in a landscape. Connectivity forests can also play a critical role for climate-change adaptation, as they can facilitate species movement in a landscape, a necessity widely predicted to occur under changing climates [35]. Assuming the development will impact only on the 1-km spatial extent of the roads, our study shows that ~374,000 ha connectivity forests and ~308,000 ha of core forests will be lost overall in PNG, if road development occurs as planned. Missing-links projects together would cause a loss of ~60,000 ha of connectivity forests and ~43,000 ha core forests, while other planned roads would cause ~310,000 and ~265,000 ha of connectivity and core-forest loss, respectively.

Some missing-link projects will have substantially higher impacts than others. Of the six missing links, the Epo-Kikori link is most critical—contributing to 39% and 49% of the loss of connectivity forest and core forest, respectively, arising from missing-link projects. This is followed by the Madang-Baiyer link (26% and 16%) and the Wau-Malalaua link (18% and 17%). Notably, the Epo-Kikori missing link will disrupt forest connectivity among three different forest ecosystems in southern PNG—mangrove forests, lowland rainforests, and montane rainforests. The Madang-Baiyer missing link will potentially destroy the last remaining forest connectivity among different protected areas and biodiversity-conservation priority areas in the Bismarck Highland region. The missing link Wau-Malalaua will reduce current forest connectivity between the southeastern rainforest and central montane rainforests in PNG.

The development of the other planned roads will particularly contribute to forest connectivity loss in northwestern and southwestern PNG (Fig 3). These road projects will crisscross the largest block of connected forest areas, which extends from Western Province in the southwest to West Sepik Province in northwest PNG. Additionally, a planned road development on the island of New Britain will crisscross both core forest and connectivity forest across the region. Most of the other road projects will reduce forest connectivity between lowland rainforests and montane rainforest both in mainland areas of PNG and PNG’s New Britain islands.

### Peatland conservation is overlooked

**Fig 4:**
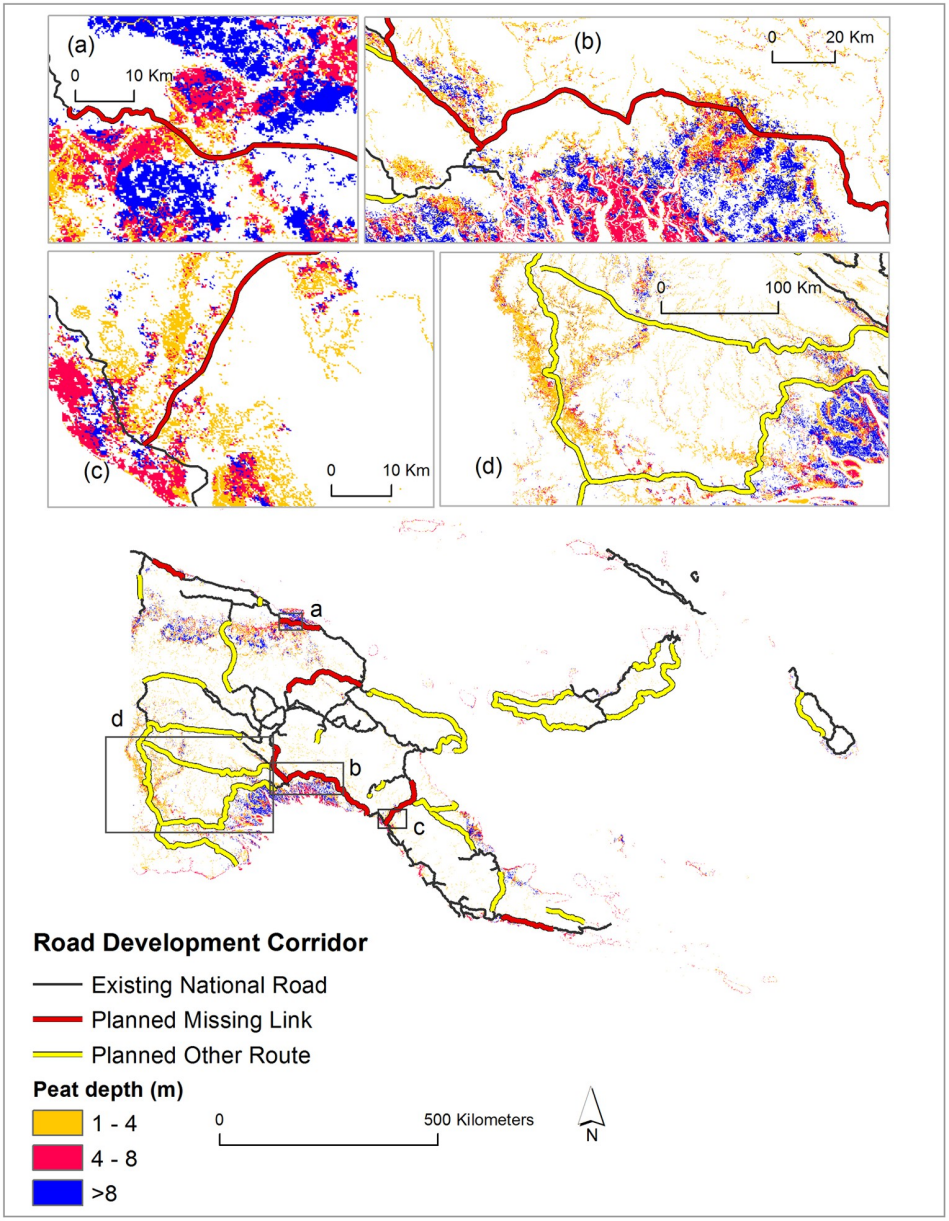
Road developments and peatlands in Papua New Guinea. (Data extracted from [27, 28]).

The planned road infrastructure expansions will impact peatlands across PNG. Considering conservatively that road development will impact only a 1-km buffer around each road, we found that ~68,000 ha peatlands will be impacted overall. Notably, 50% of these are deep peatlands (>4m deep) and 15% are very deep peatlands (>9m deep), which have extremely high carbon storage values (Fig 4).

Overall, 333 km of the planned roads will traverse peatlands. Of the ~1,061 km of nationally prioritized missing-link projects, ~78 km will traverse peatlands impacting ~16,000 ha of peatland habitats. The Epo-Kikori missing link project would be the most risky, traversing 34 km of peatlands, followed by the Bogia-Angoram (17 km) and Wau-Malalaua (8 km) missing links. The Bogia-Angoram missing-link would dissect a large block of very deep peatland (>9m deep) impacting ~1,000 ha in this category (Fig 4a) while the Epo-Kikori missing link would dissect one of the largest continuous blocks of peatlands in PNG, impacting ~7,000 ha of peatlands in total (Fig 4b). The Wau-Malalaua missing link would impact ~1,700 ha of peatlands in the Malalau region, which is a continuation of the region’s largest peatland block (Fig 4c). Furthermore, 256 km of other low-priority planned road projects would traverse peatlands, impacting ~52,000 ha overall, particularly in the Western Province of PNG (Fig 4d).

This development of road corridors in peatland would have at least three immediate effects on environmental, economic, and social contexts in the region: (i) Road construction and associated disturbances in the peatland would emit a substantial amount of greenhouse gases. As carbon storage in peatlands is high, it would also make remaining peatlands vulnerable to future disturbances such as fire, facilitating further emissions that would vary with peat depth. (ii) Road construction and subsequent maintenance costs of road segments in peatlands will be high. This necessitates more expenses in the absence of sufficient future investment for road maintenance. The current road network in PNG is in poor condition due to the lack of investment in road maintenance [10], mirroring the likely future condition of these roads. (iii) Road construction and associated disturbances can change peatland hydrology, potentially draining peatlands and lowering their water table, thereby increasing peat fires and peat oxidation, both major sources of carbon emissions [36]. In Southeast Asia, the burning of peatlands during droughts has created regional environmental catastrophes, including extremely degraded air quality from haze [37]. The same could happen in PNG.

### New deforestation frontiers

**Fig 5:**
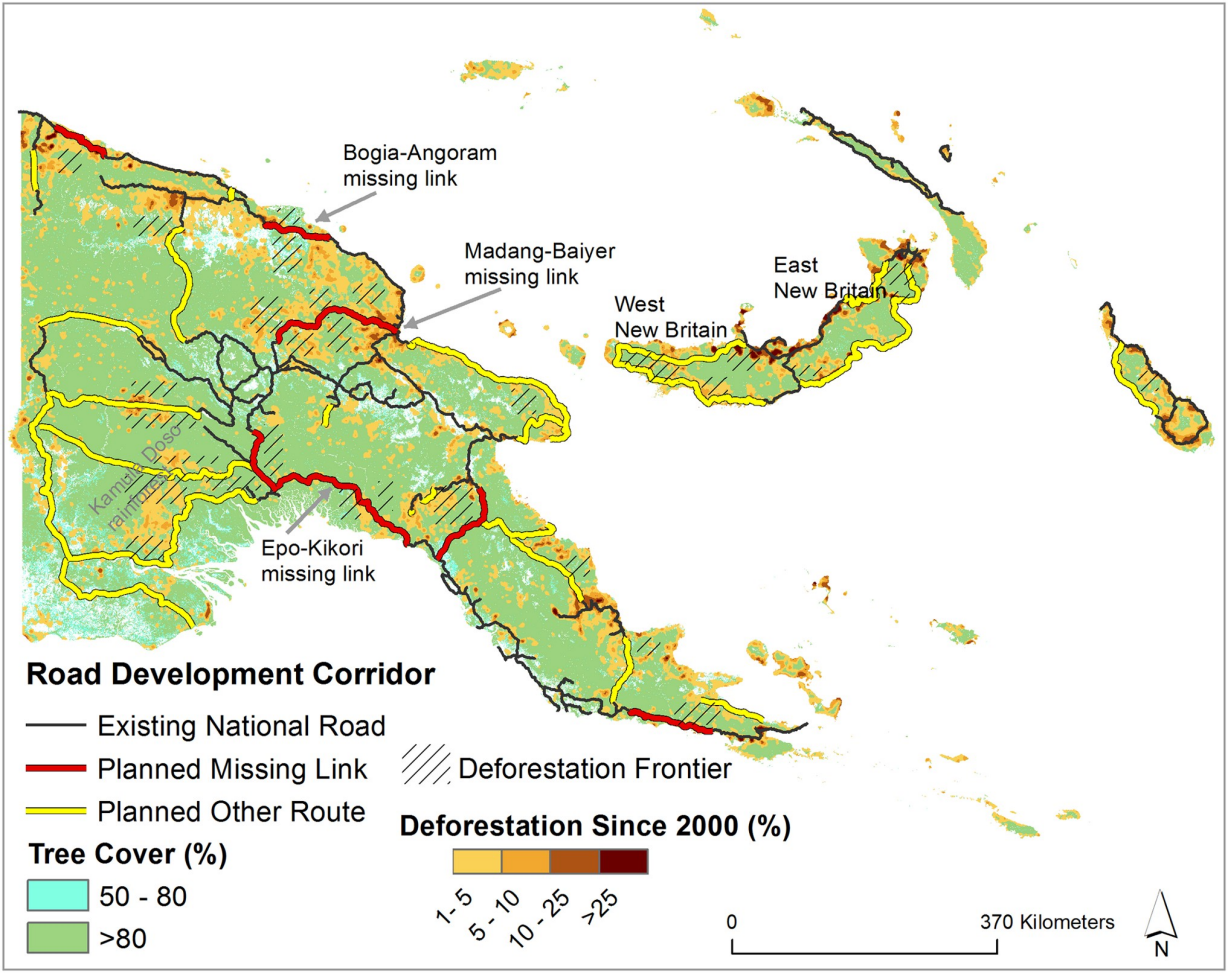
Planned road development and potential deforestation frontiers in Papua New Guinea. Deforestation rate refers to the percentage of the area deforested since 2000 within a 3-km radius of any pixel.

Several new deforestation frontiers are highly likely to develop across PNG, should road-expansion projects occur as planned. We assessed local deforestation rates across PNG since 2000 and found that deforestation is highly spatially contagious along current road infrastructure (Fig 5), as is typical across the tropics [5, 6, 8]. Hence, new deforestation frontiers are likely to arise in current forested areas that are (1) in close proximity to planned missing links or other proposed roads, and (2) located close to current deforestation zones. Our analyses suggest five new deforestation frontiers are likely to arise if missing links and other proposed road projects are implemented as planned:

1. In Madang, current deforestation frontiers are being driven by logging and mining. The planned Madang-Baiyer missing link will increase current deforestation frontiers in remaining relatively non-degraded forests (canopy cover >80%) on both sides of the missing link (Fig 5). The current deforestation drivers, legal and illegal logging and further mining exploration, are likely to infiltrate into remaining undisturbed forests and provoke these new deforestation frontiers.
2. In East New Britain and West New Britain, current deforestation frontiers are expanding rapidly due to large-scale oil-palm plantations along existing national roads. The proposed road projects in this region will be linked with existing national roads and will eventually provide access to remaining undisturbed forests, which are currently inaccessible by high-quality roads (Fig 5) that are a precondition for effective oil palm harvesting. The PNG government is working with different stakeholders to increase oil palm plantations in the region. The proposed roads appear to be a supporting initiative for expansion of oil palm plantations in this region and will facilitate imminent deforestation there (Fig 5).
3. Current deforestation frontiers in the Bogia-Angoram missing link region are being driven by logging and mining. Most forests in this area are being converted to other land uses and remaining forests are in a state of degradation (50–80% canopy cover). The planned missing-link project in this region which aims to connect existing national roads (Fig 5) would likely initiate deforestation frontiers that are near wetlands in the Angoram region.
4. Remaining forests in the Epo-Kikori missing-link area are relatively undisturbed (canopy cover >80%). Numerous deforestation frontiers are likely to arise in this region if this project proceeds as planned, particularly facilitated by mining in the Gulf Province. Several mining-exploration projects are already present in the region along the planned Epo-Kikori missing link, which increases the likelihood of imminent deforestation in this region were roads developed as planned (Fig 5).
5. The current road development plan in PNG consists of a number of road projects, such as the Epo-Kikori missing link and other planned projects in the remote western provinces, that will degrade the Kamula Doso rainforest (Fig 5). This rainforest contains one of the highest carbon-storage levels of any lowland rainforest in PNG. Moreover, it is the largest track of remaining lowland rainforest in PNG. If development proceeds as planned, the forest in this region would suffer from deforestation and degradation, particularly under the mounting threat of commercial logging fuelled by foreign investors.

### Landslides and impeded drainage

**Fig 6:**
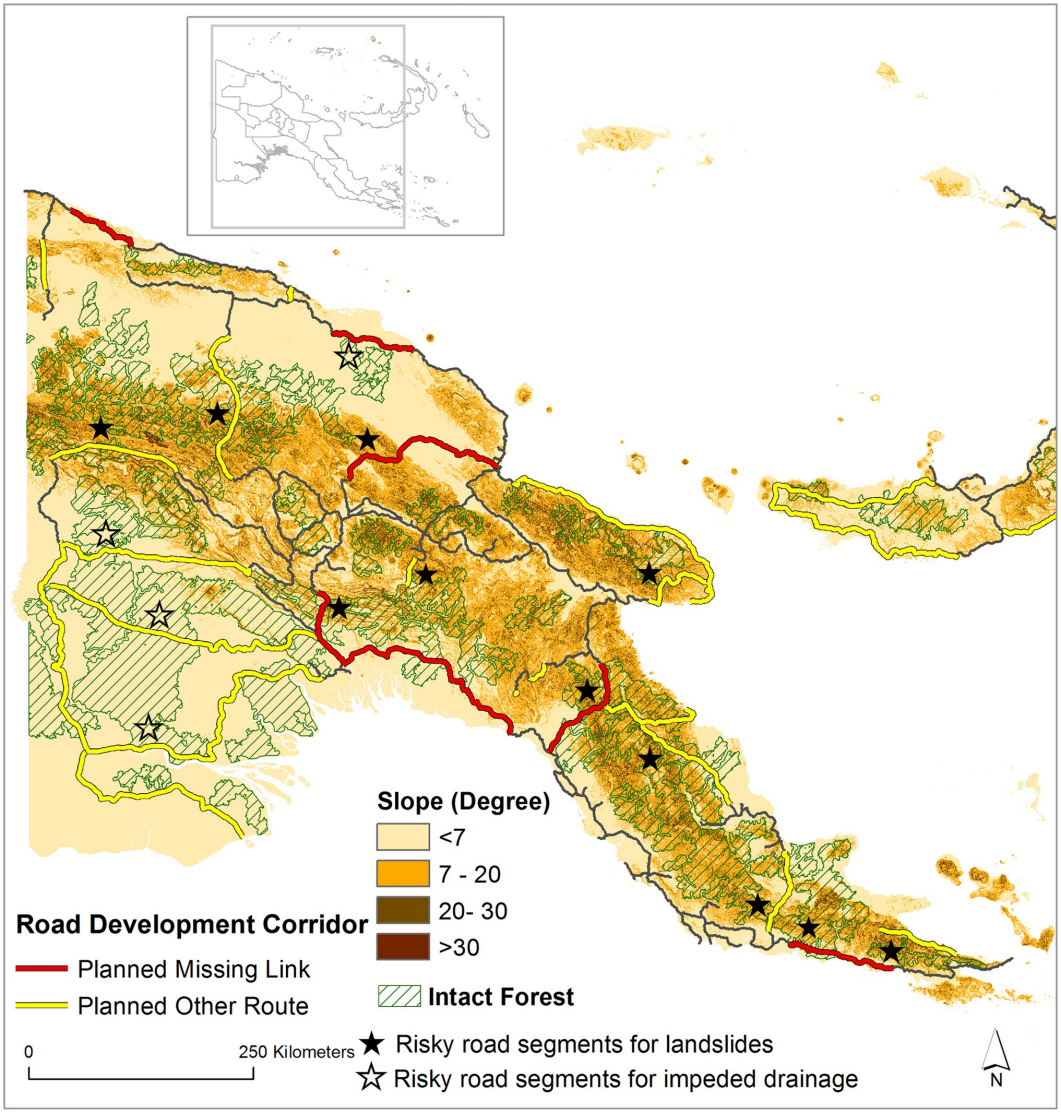
Planned road expansion and imminent frontiers for landslides and impeded drainage in Papua New Guinea. Intact forest (data from Potapov et al. [40]) indicates an area of more than 500 km^2^ without any significant sign of human activity.

**Fig 7:**
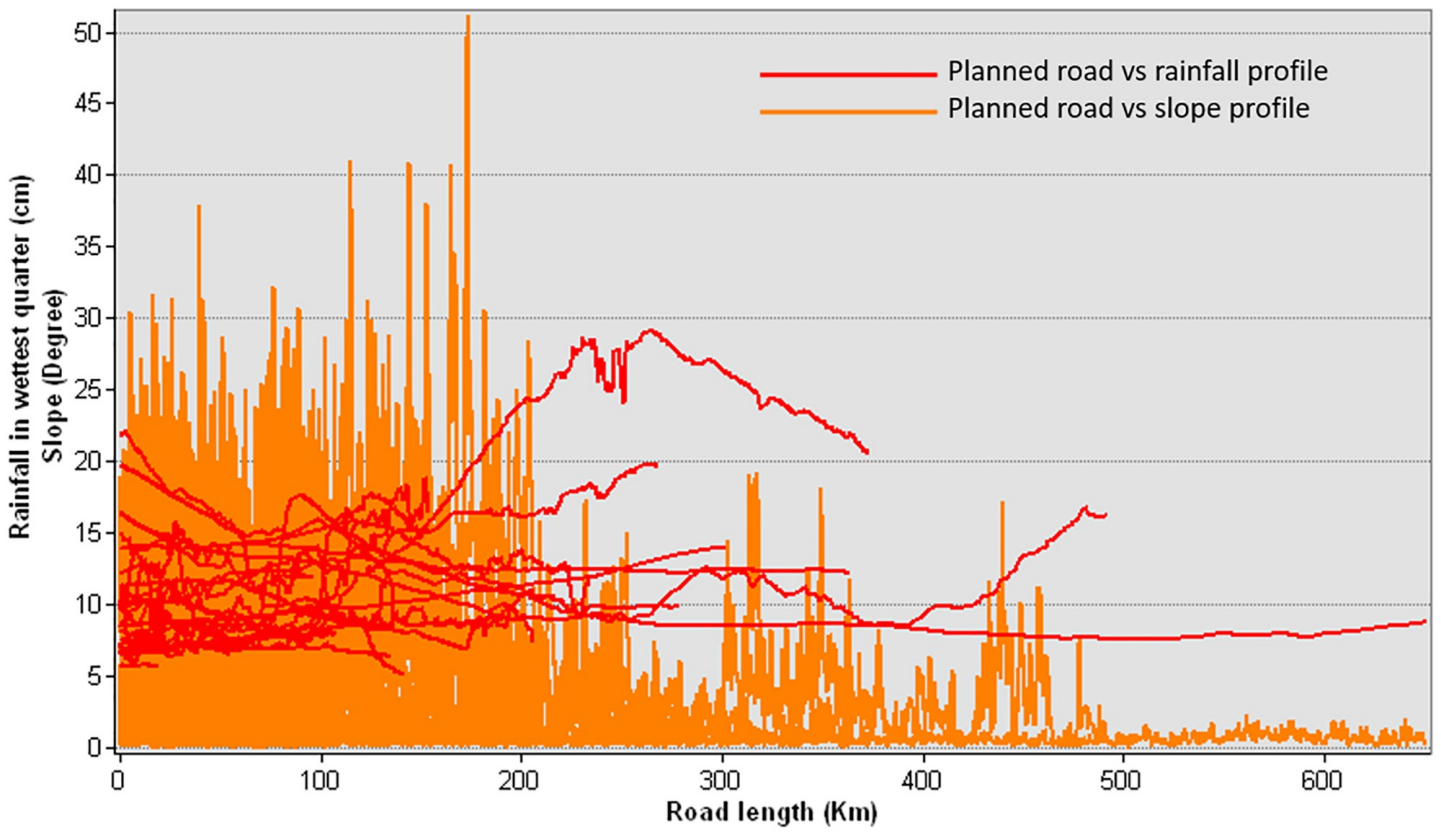
Planned roads with respect to topographic slope and total rainfall in wettest quarter.

In a country as steep and dissected as PNG, planned road expansion projects will increase landslides and impede drainage conditions. By mapping planned roads against rainfall and topographic slope (Figs 6 and 7), our results suggest that several planned road segments will traverse intact forests that are highly vulnerable to landslides (>20 degrees slope and >1,500 mm of rainfall in the wettest quarter). We also mapped areas vulnerable to impeded drainage conditions (<7 degrees slope and >1000 mm of rainfall in the wettest quarter), which can lead to localized flooding and potholing of roads. Landslides are likely to increase in many steep sites in PNG where new roads are being built, as observed elsewhere in the tropics [5]. Additionally, PNG suffers from frequent earthquakes, which triggers landslides, some of which are massive. The risk of landslides during earthquakes will be exacerbated by road construction, particularly in steep terrain and deforested areas.

In general, many missing-link projects would contribute to landslides whereas other planned road projects would promote impeded drainage (Figs 6 and 7). The missing link Epo-Kikori appears the most risky for facilitating increased landslides, followed by the Madang-Baiyer missing link, and the Wau-Malalau missing link. Other planned roads in western PNG also appear at risk of impeded drainage, including the one that traverses the largest block of intact lowland rainforest in PNG (Figs 6 and 7). This drainage impediment can facilitate large-scale die-offs of forest and other vegetation by facilitating forest flooding in the wet season [5].

Considering the risks of roading in steep terrain, logging guidelines in PNG prohibit logging in areas with >30 degrees slope [38]. In neighboring Indonesian New Guinea, however, logging is prohibilted in areas with >20 degrees slope [39]. In PNG, the current plan of building roads in steep areas (Figs 6 and 7) will clearly require roading, earthworks, treefelling, and soil damage that is contradictory to PNG logging regulations. Landslides are already increasing in various sites in PNG where new roads have been built. Beyond landslides, these road segments will increase soil erosion and stream sedimentation, with potentially serious impacts on water quality, the stability of water flows, the economic viability of fisheries, and downstream flood risk [5].

## Discussion

### Unique biodiversity values are ignored

PNG is a land of exceptional species endemism and high biodiversity—containing ~5% of all global vertebrate species [1]. Planned road-expansion projects would clearly have a wide range of impacts on the biodiversity and environmental stability of PNG. This would happen in many different forms as our study suggests—via deforestation, forest fragmentation, dividing key biodiversity-conservation areas and protected areas, reducing current forest connectivity, and degrading forest-interior habitats (Figs 2, 3 and 5). All these consequences of road development have been reported from tropical forests elsewhere, such as in nearby Indonesian Papua and West Papua [22], Borneo [11], and the Amazon [8, 18, 41].

The planned roads would create dire consequences for iconic bird of paradise species, penetrating their habitats in a number of places such as the Bismarck Highland and Jimi Valley areas through the Madang-Baiyer missing link. Most species of birds of paradise are endemic to the rainforests of PNG [42]. Apart from direct habitat loss and habitat isolation of these iconic species, planned roads would increase access to hunters that have taken a heavy toll on many species.

Many wildlife species in PNG—including several endangered species facing high extinction risks from overhunting and habitat loss—would become more vulnerable to local extinction following planned road construction. A widespread decline of long-beaked echidna (*Zaglossus bartoni*) and Goodfellow’s tree-kangaroo (*Dendrolagus goodfellowi*) in PNG has been reported due to over-hunting, deforestation, and forest degradation in recent years [1]. Road construction in their key habitats—such as the Madang-Baiyer missing links and planned roads in the central-highland region—would exacerbate those losses. Road expansion and associated increases in hunting have devastated vulnerable wildlife elsewhere in the world, including a decline of over 60% of African forests elephants (*Loxodonta cyclotis*) in the last decade [43]. Sumatran orangutans (*Pongo abelii*) and Tapanuli orangutans (*P*. *tapanuliensis*) in Indonesia are both critically imperilled by road and infrastructure proliferation in their remaining habitat [12, 44, 45].

Habitat conservation of the endangered Matchie’s tree kangaroo (*Dendrolagus matschiei*) and other iconic specices has been effective in the YUS conservation area [34]. However, planned roads would dissect the YUS area (Fig 2) and imperil both the Matchie’s tree kangaroo and other vulnerable wildlife, such as the range-restricted emperor bird-of-paradise (*Paradisaea guilielmi*) and Huon astrapia (*Astrapia rothschildi*).

Planned roads would sharply reduce the resilience of many species to climate change, via habitat fragmentation and losses of forest connectivity (Fig 3) that limit the capacity of species to shifts to more suitable habitat [35]. Forest fragmentation alters many microclimatic conditions, leading to higher air temperatures and wind disturbances and lower soil moisture than in intact forest [18]. Such changes would worsen the capacity of forests to buffer climate change, particularly with warming and drying conditions. Fragenented forests also facilitate higher invasive and non-endemic species abundances and diverse changes in plant and animal communities [46, 47].

Current road-development plans in PNG would undermine ambitions to rapidly increase the number of effective protected areas [48]. Roading plans would also degrade many key biodiversity-conservation areas (Fig 2) that are strong candidates for immediate conservation to reach national protected-areas targets [49]. Roads within and in the periphery of protected areas can also sharply degrade habitat quality [50], as demonstrated in nearby Indonesia [51]. Beyond risks to protected areas, new roads would open up currently undisturbed protected areas for deforestation and forest degradation, such as those in the Hunstein Range in East Sepik Province.

### Risks of forest exploitation and conversion

The present rate of deforestation and forest degradation in PNG is the highest in the country’s history. These impacts are largely concentrated in lowland forests (Fig 5), facilitated by growing road accessibility and commercial logging and mining [1, 3]. It is anticipated that timber stocks in most lowland forests in PNG will decline sooner than expected due to widespread logging and forest degradation [4].

**Fig 8:**
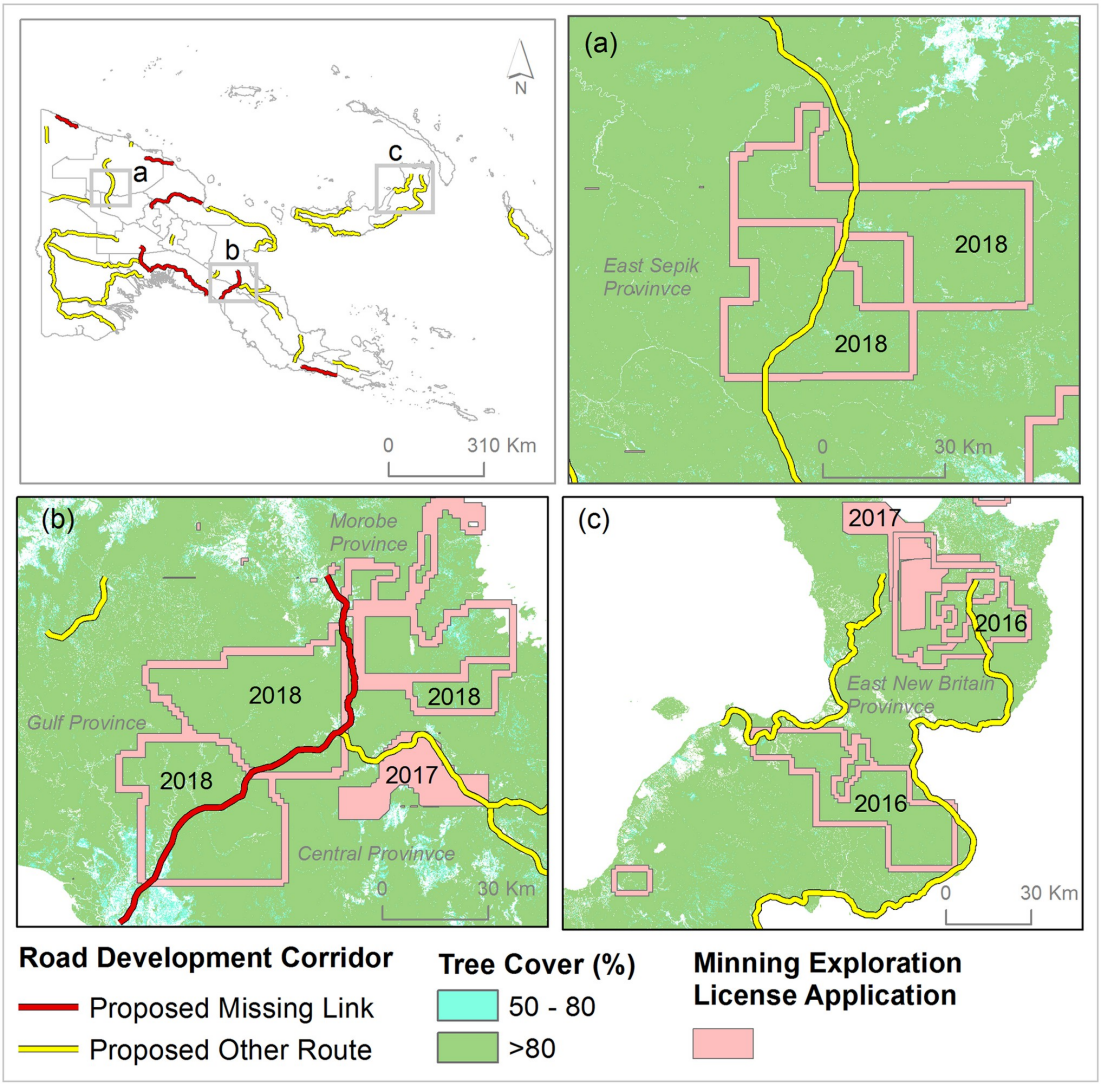
Planned road expansion and recent mining-license applications. The map indicates the year of application for mining-exploration licenses. These applications are under varying stages of processing for approval.

Conversely, the rate of deforestation and forest degradation in highland forests is much lower than in the lowlands (Fig 5), mainly because of the absence of large-scale road networks. Many planned road segments such as the Madang–Baiyer missing link, part of the Epo-Kikori missing link, and the Wau-Malalaua missing link (Figs 1 and 5) will greatly increase accessibility to upland forests, promoting wider exploitation in line with government schemes to increase logging, mining and large-scale oil-palm plantations [2, 10]. In fact, many new mining-exploration licenses have already been initiated along planned road routes (Fig 8) [32], especially since 2018 [32, 52].

Further expansion of oil palm plantations will degrade remaining lowland forests in both mainland PNG and its islands [53]. A major scandal in PNG has been the “Special Agricultural and Business Leases” (SABLs), which allowed clear-felling of forests for large-scale agricultural development, particularly oil-palm plantations [1]. In most cases, companies holding SABLs did not establish plantations but used the SABLs to facilitate land grabbing and bypass forestry regulations to conduct unregulated logging [53]. New leases under the SABL policy have now been abandoned because of recurring allegations of land grabbing and substantial irregularities [54]. Several planned road projects in provinces such as West Sepik, East Sepik, West New Britain, and East New Britain are a strong indicator of further schemes to expand oil palm in the region [2, 10], placing many lowland forests in severe danger. An example is the Managalas conservation area, which was recently declared to protect it from logging and industry-scale agriculture [25], but which could be opened up for mining by planned construction of a major new road (Fig 2).

### Low-carbon development and SDGs

Low-carbon development is a national development strategy that aims for low greenhouse gas emission or a national development plan resilient to climate change [55]. The current road development plan in PNG effectively ignores low-carbon development, not taking into consideration the huge carbon stock in peatlands (Fig 4) and hyperdense rainforests (Fig 5). PNG contains the second largest peatland in Asia after Indonesia, with ~45,000 km^2^ of peatlands [27]. These peatlands are an important global carbon pool [56]. Heavy logging is also a major source of CO_2_ emissions. The current road development agenda in the peatlands and hyperdense rainforest areas will release substantial carbon to the atmosphere and set the stage for future emissions.

PNG has put substantial efforts into the development of a REDD (Reduced Emissions from Deforestation and Forest Degradation) program [57] and aspires to achieve carbon-neutral development by 2050 [58]. The current road development plan in peatlands and high-carbon rainforests, and the major increases in forest loss and fragmentation they would foment, are clearly inconsistent with such commitments. Similarly, road development followed by clear-felling of intact forests for industial-scale development (e.g. SABLs [59]) are contrary to PNG’s commitment to REDD and low-carbon development.

With its planned road development, PNG would also not honor its international commitments towards the U.N. Sustainable Development Goals (SDGs). PNG is enthusiastically working with the global community to achieve SDGs [60], and in many cases is leading Pacific nations to progress towards the SDGs [60]. If road development occurs as currently planned, it will have substantial impacts on terrestrial ecosystems, biodiversity, and carbon storage. PNG’s vision to sustainable development is clear but lacks an ecosystem-based development pathway [61]. Current road-development plans would undermine key SDG aspirations and targets.

### Risks of economic impacts

Surging long-term debt is an alarming possibility in the aftermath of planned road development in PNG. Road construction and maintenance costs are high in PNG because of difficult topography and high rainfall, the remoteness and high transportation costs for getting material to worksites, and substantial corruption costs from road-construction cartels. A number of planned road segments are set to cross extreme terrain, contributing to even higher construction costs (Figs 6 and 7). As our study reports, these segments would be extremely vulnerable to landslides during heavy rainfall events and earthquakes. These impacts would extend beyond environmental to social and economic sectors. For instance, large-scale landslides and flash floods in PNG [62] and Papua, Indonesia [63] exacerbated by road construction recently cost millions of dollars to their national economies. Along with high road-construction costs, PNG would need major budget allocations for maintenance to keep the roads in useable condition. However, only 33% of the current national roads in PNG are in useable condition [64], with the remainder in very poor condition due to inadequate funding for maintenance [10].

## Conclusions and recommendations

PNG needs to upgrade and establish key road segments to advance its current development agenda. However, such projects need to be economically feasible, socially equitable and realistic, and environmentally sustainable. Our study suggests that current plans for many road segments have not adequately considered the unique environmental values, economic risks, and social settings of PNG. Many road segments will create large overall risks and potentially return only marginal benefits. Therefore, we suggest the following based on our informed and detailed analysis:

- Cancelling the planned road segments that will pass through protected areas, key habitats of endangered and iconic wildlife species, and major peatlands. These specifically include the Madang-Baiyer missing link, Bogia-Angoram missing link, Wau-Malalaua missing link, the planned road in East Sepik Province connecting Pogera and Ambunti, and the planned roads dissecting the YUS and Managalas conservation areas.
- Road segments set to bisect interior-forest areas and biodiversity conservation-priority areas will diminish forest connectivity and create imminent deforestation frontiers. These should be recognized as having particularly high environmental risks. Special safeguards, including increased law enforcement, impact-mitigation strategies, and initiatives to expand new protected areas along road routes, are highly advisable.
- Both missing links and proposed road segments identified to occur at >30 degrees slope in intact forest areas should be cancelled or greatly minimized, following the logging guidelines of PNG, which prohibits logging of such steep slopes. Such roads will have exceptionally high construction and maintenance costs, and severe environmental risks for local and downstream ecosystems and landowners.
- Planned roads in the periphery of the Kamula Doso rainforest—the missing link connecting Kikori and Kagua (part of Epo-Kikori missing link), and other road projects in Western Province—should be re-evaluated considering their high ecological and carbon-emissions risks. These areas might be better protected under the REDD+ initiative.
- Investment by foreign financiers should largely focus on upgrades and maintenance of existing roads, rather than new road construction. The Australian government’s current policy is exemplary—prioritizing investment on road maintenance in PNG rather than new road building, to keep the national road network in workable condition [64]. This is an urgent and realistic priority, as some 67% of current national roads in PNG are in very poor condition [64].
- International support is direly needed to help PNG explore more sustainable development pathways. At present, heavy foreign investment, especially from China [65–70], is promoting many high-risk and potentially predatory development projects in PNG and elsewhere in the Asia-Pacific region.
